# Supplementary material for: Single cell transcriptomics identifies a signaling network coordinating endoderm and mesoderm diversification during foregut organogenesis
Source: Nat Commun. 2020 Aug 27;11:4158. doi: 10.1038/s41467-020-17968-x (PMC7453027; doi:10.1038/s41467-020-17968-x)
Supplement: Supplementary file 1 — Supplementary Information [file 41467_2020_17968_MOESM1_ESM.pdf]

Supplementary Information: Nature Communications

Single cell transcriptomics identifies a signaling network coordinating endoderm and mesoderm diversification during foregut organogenesis

Lu Han[#1], Praneet Chaturvedi[#1], Keishi Kishimoto[#1,2,3], Hiroyuki Koike[#4], Talia Nasr[1], Kentaro Iwasawa[4], Kirsten Giesbrecht[4], Phillip C Witcher[1], Alexandra Eicher[1], Lauren Haines[1], Yarim Lee[1], John M Shannon[5], Mitsuru Morimoto[2, 3], James M Wells[1], Takanori Takebe[4], Aaron M Zorn[*1,3]

[1]Center for Stem Cell & Organoid Medicine (CuSTOM), Perinatal Institute, Division of Developmental Biology, Cincinnati Children's Hospital. Department of Pediatrics, University of Cincinnati, College of Medicine. Cincinnati, OH, 45229, USA. [2]Laboratory for Lung Development, RIKEN Center for Biosystems Dynamics Research (BDR), Kobe, 650-0047, Japan, [3]CuSTOM-RIKEN BDR collaborative laboratory, Cincinnati Children's Hospital, Cincinnati, OH, USA. Divisions of [4]Gastroenterology and [5]Pulmonary Biology, Cincinnati Children's Hospital. Department of Pediatrics, University of Cincinnati, College of Medicine. Cincinnati, OH, 45229, USA.

# These authors contributed equally.

* correspondence: Aaron.zorn@cchmc.org

Contents:

Supplementary Figures 1-12
Supplementary Tables 1-2

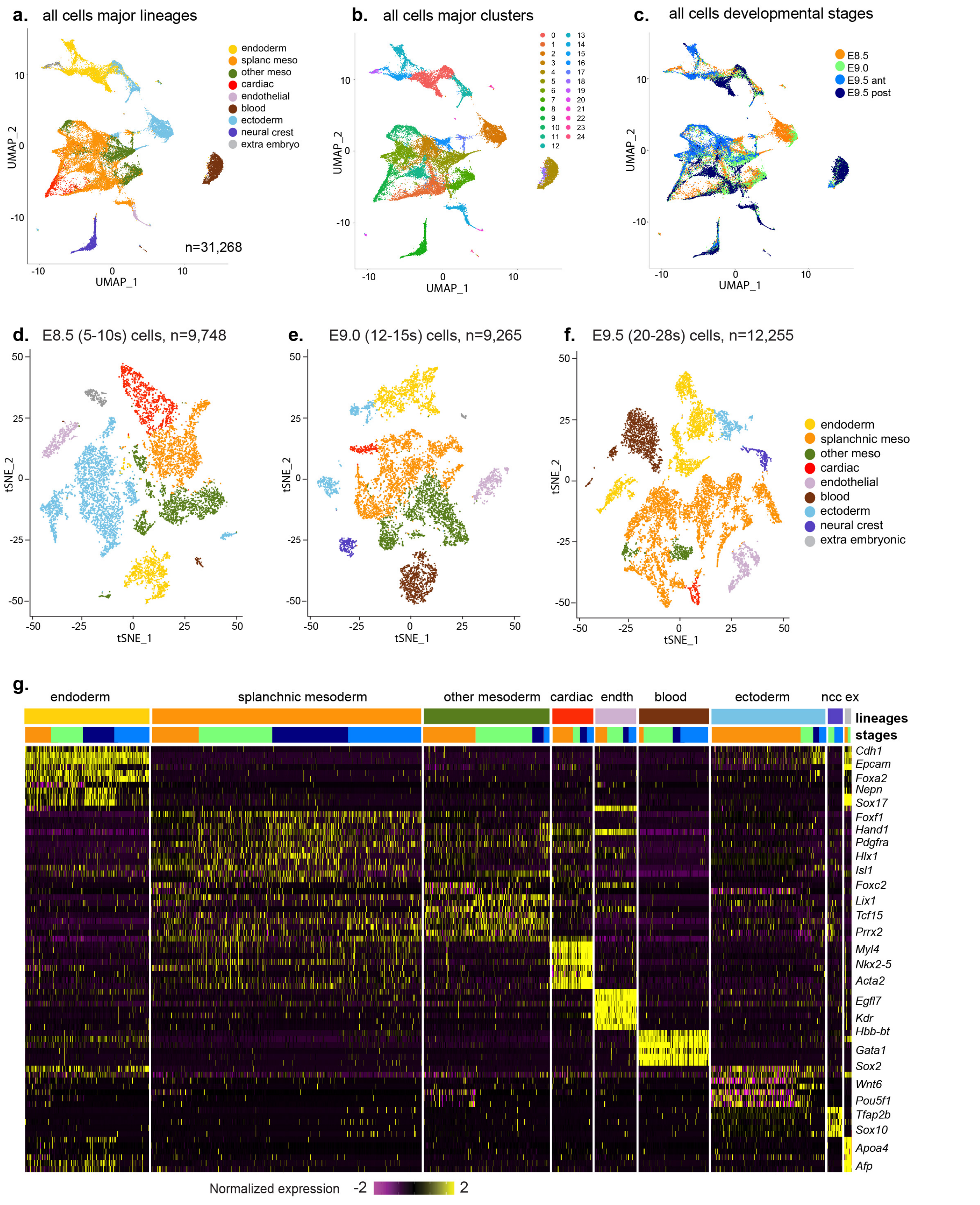

Supplementary Figure 1. Defining major cell lineages. a, UMAP of single cells from all stages with major lineage annotated by known marker genes. **b,** UMAP of all cells from all stages with computationally assigned cell clusters based on transcriptome similarity. **c,** UMAP of all cells from all stages color by stages and regions. **d-f**, tSNE map of single cells from each stage annotated by major lineages at E8.5 in d), E9.0 in e) and E9.5 in f). **g**, Gene expression heatmap of selected markers in individual cells across different lineages and stages. Min-Max row normalized. Relevant to Fig. 1.

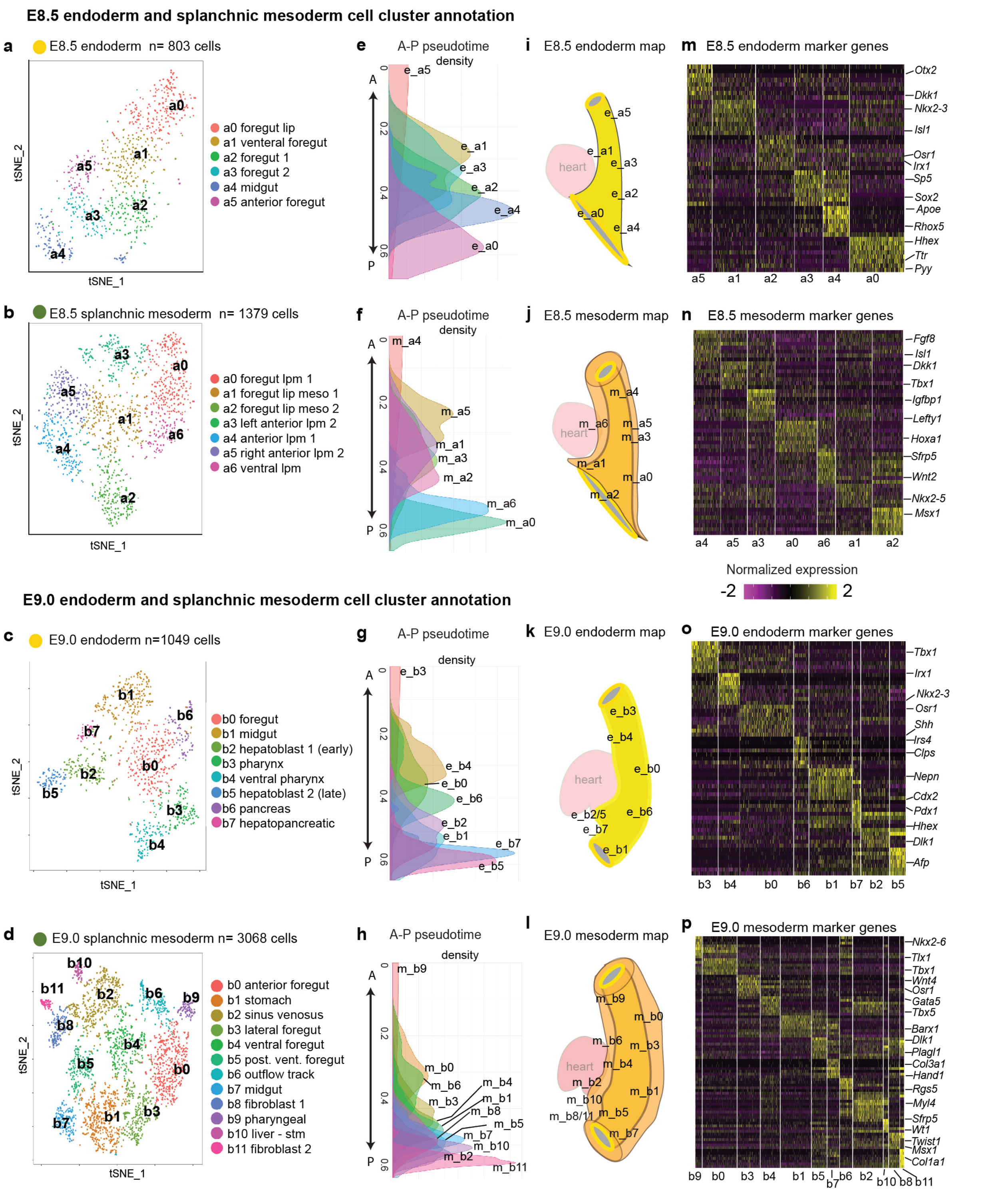

Supplementary Figure 2. Annotation of E8.5 and E9.0 DE and SM lineages. a-d, t-SNE plot of E8.5 DE (**a**), E8.5 SM (**b**), E9.0 DE (**c**) and E9.0 SM cells (**d**) annotations. E8.5 clusters are designated as a, E9.0 as b, and E9.5 as c. **e-h,** Pseudo-spatial ordering of E8.5 DE (**e**), E8.5 SM (**f**), E9.0 DE (**g**) and E9.0 SM cells (**h**) along the anterior-posterior (A-P) axis of the gut tube. **i-l,** Schematics of the mouse embryonic foregut showing the predicted location of E8.5 DE (**i**), E8.5 SM (**j**), E9.0 DE (**k**) and E9.0 SM (**l**) cell types mapped onto the endoderm (yellow) and mesoderm (orange)**. m-p,** Heatmap of selected marker gene expression (min-max row normalized) in individual cells across different clusters at E8.5 DE (**m**), E8.5 SM (**n**), E9.0 DE (**0**) and E9.0 SM (**p**). Relevant to Fig. 1.

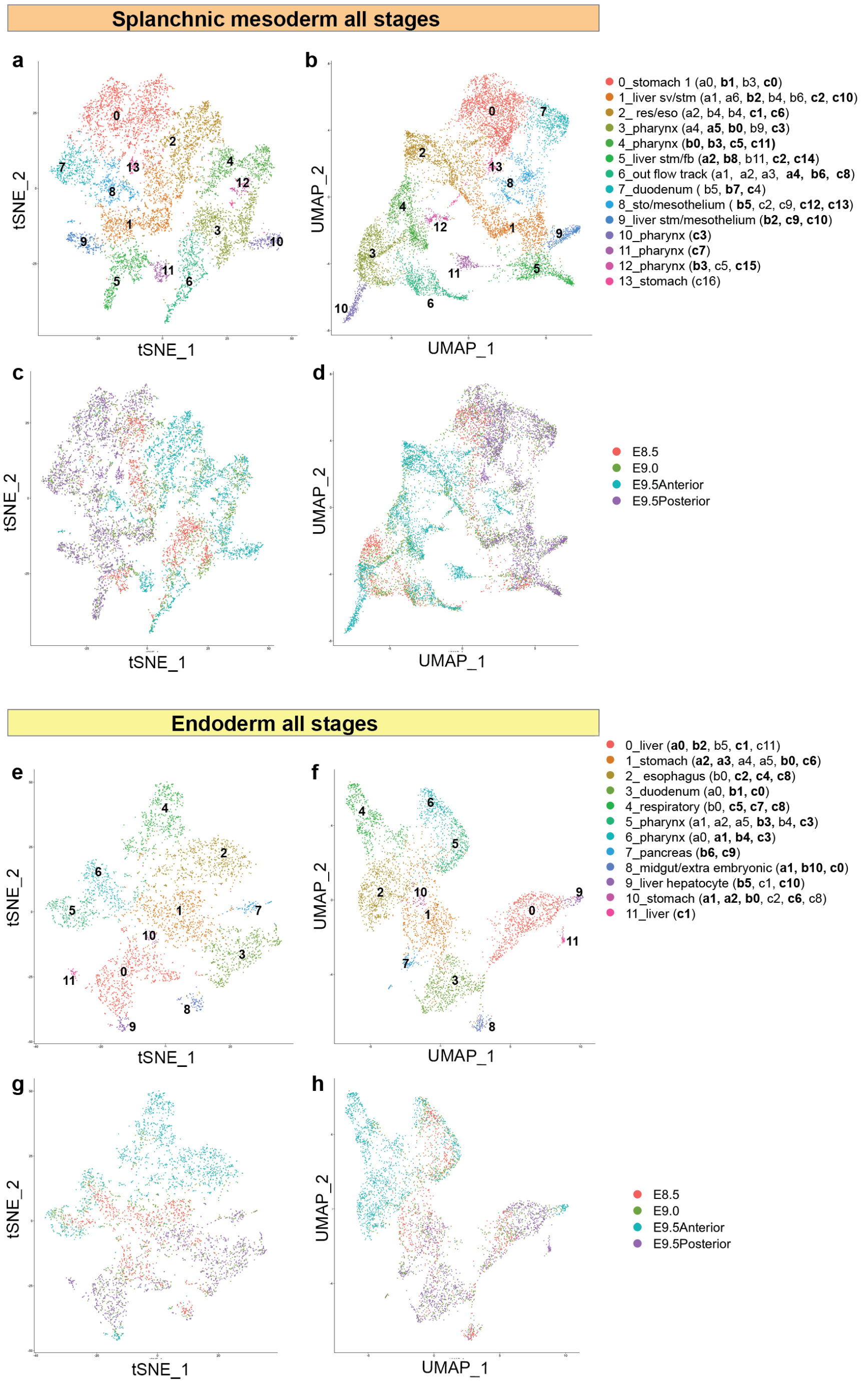

Supplementary Figure 3. Integrated analysis of DE and SM cells. a-d, tSNE and UMAP visualization of all SM cells from all stages annotated by major lineages (a,b) and stages (c,d) **e-h**, tSNE and UMAP visualization of all DE cells from all stages annotated by major lineages (e,f) and stage (g,h). The stage-specific annotations making major contributions to each integrated cluster are indicated in brackets. E8.5 cells = a_clusters, E9.0 cells = b_ clusters and E9.5 = c_clusters. Relevant to Fig.1

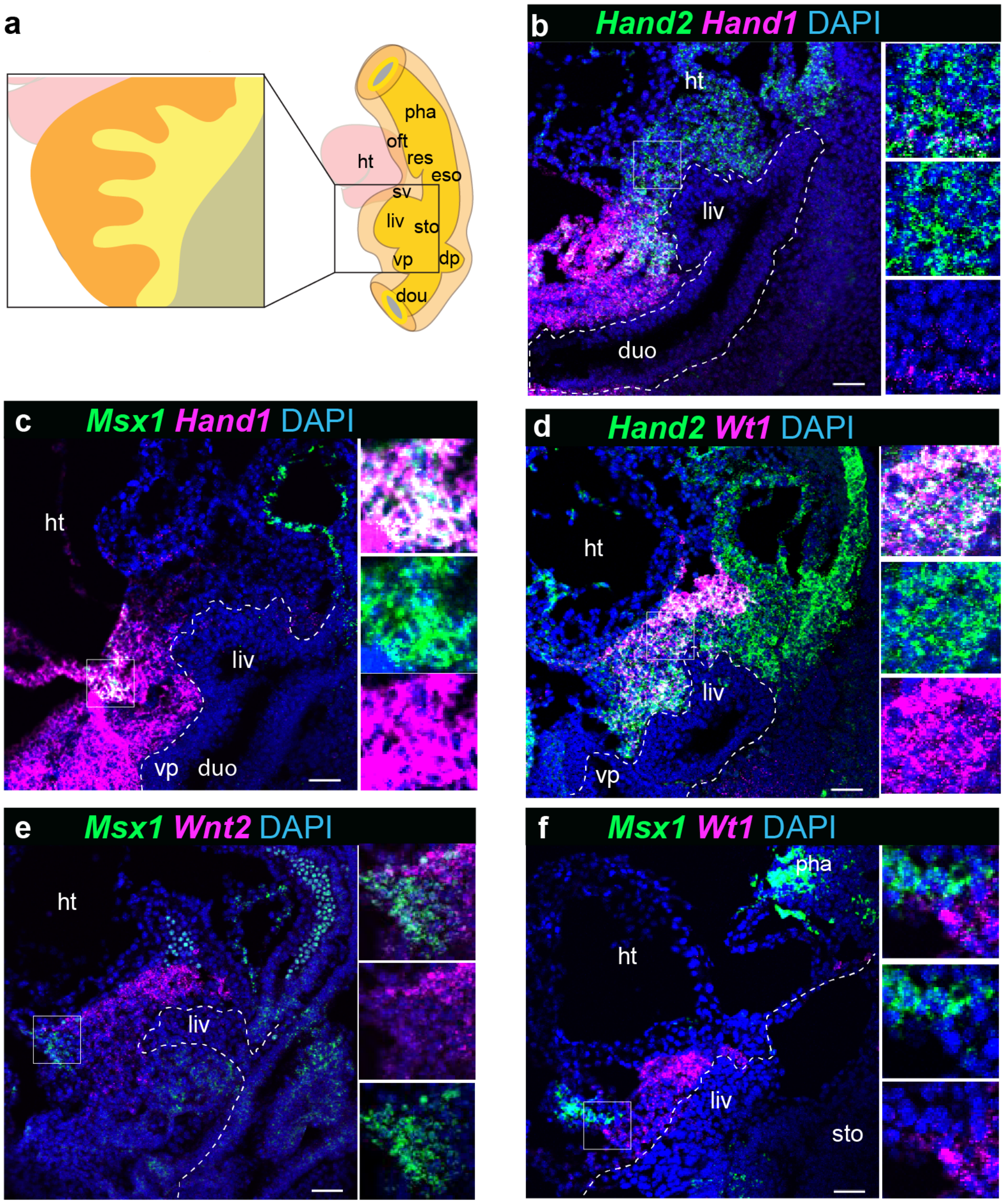

Supplementary Figure 4. Validation of liver mesenchyme subtypes. a, Schematic of a mouse embryonic foregut at E9.5. Magnified panel shows sagittal section of the liver bud. **b-f,** RNA-scope *in situ* detection of mesoderm markers on fixed frozen sagittal sections from E9.5 mouse embryos. duo; duodenum, dp; dorsal pancreas, eso; esophagus, ht; heart, liv; liver, oft, outflow tract, pha pharynx, res; respiratory, stm; septum transversum, mesenchyme, sto; stomach, sv; sinus venosus, vp; ventral pancreas. Scale bar 50μm. Insets show high magnification of boxed region with merge and separate channels. n=2/2 embryos for each probe combination. Relevant to Fig. 2.

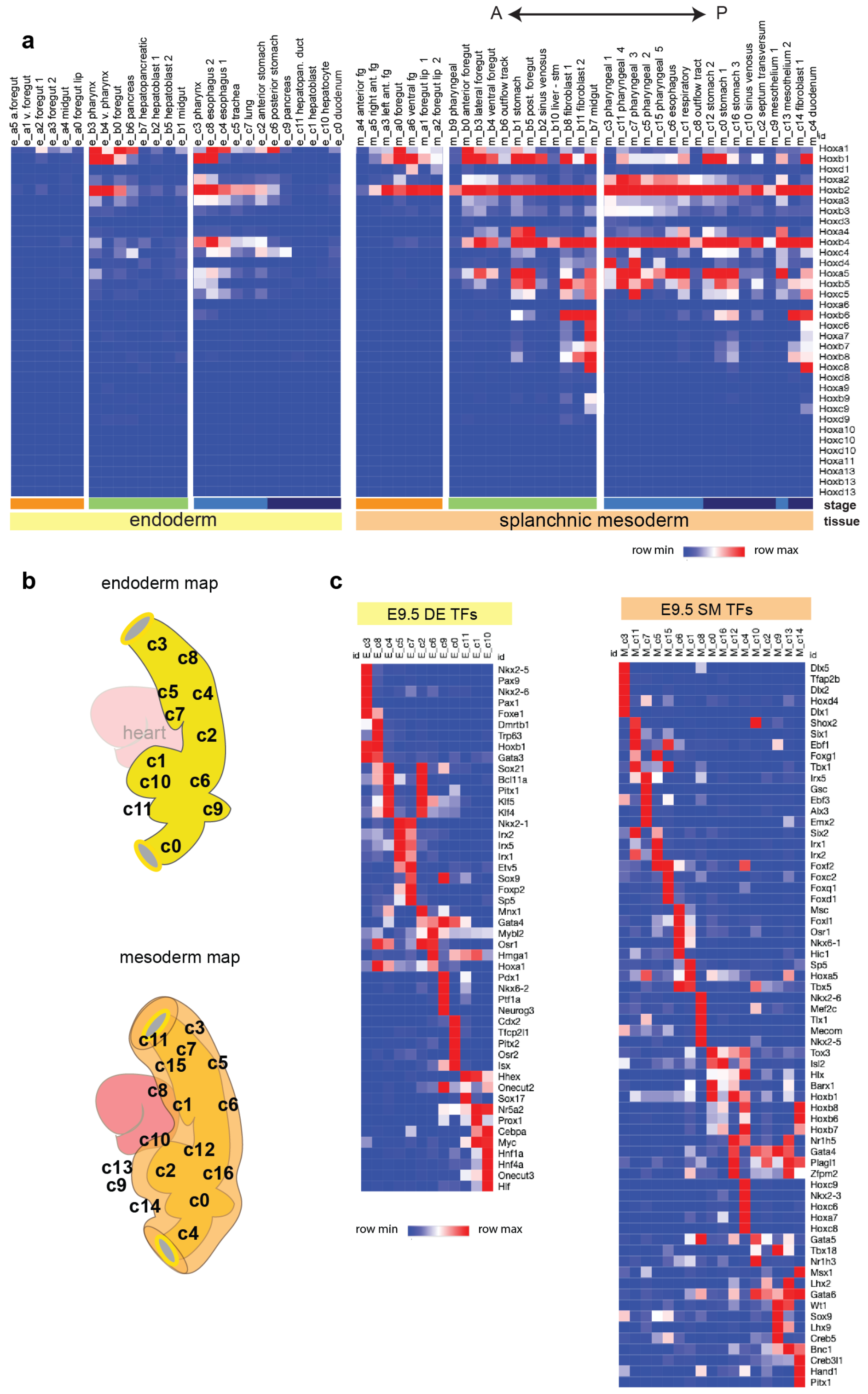

Supplementary Figure 5. Co-linear *Hox* gene expression and Transcription factor code. a, Heatmaps of average *Hox* gene expression in different DE and SM clusters arranged along the anterior (A) - posterior (P) axis. Annotations are; E8.5 = a_clusters, E9.0 = b_clusters and E9.5 = c_clusters. **b**, Inferred location of cell clusters in the foregut endoderm and mesoderm. **c**, Transcription factor code. Heatmap showing the average expression of top five distinguishing transcription factor (TFs) differential expressions across E9.5 DE and SM populations. a, anterior; fg, foregut; post, posterior; v, ventral; stm, septum transversum mesenchyme.

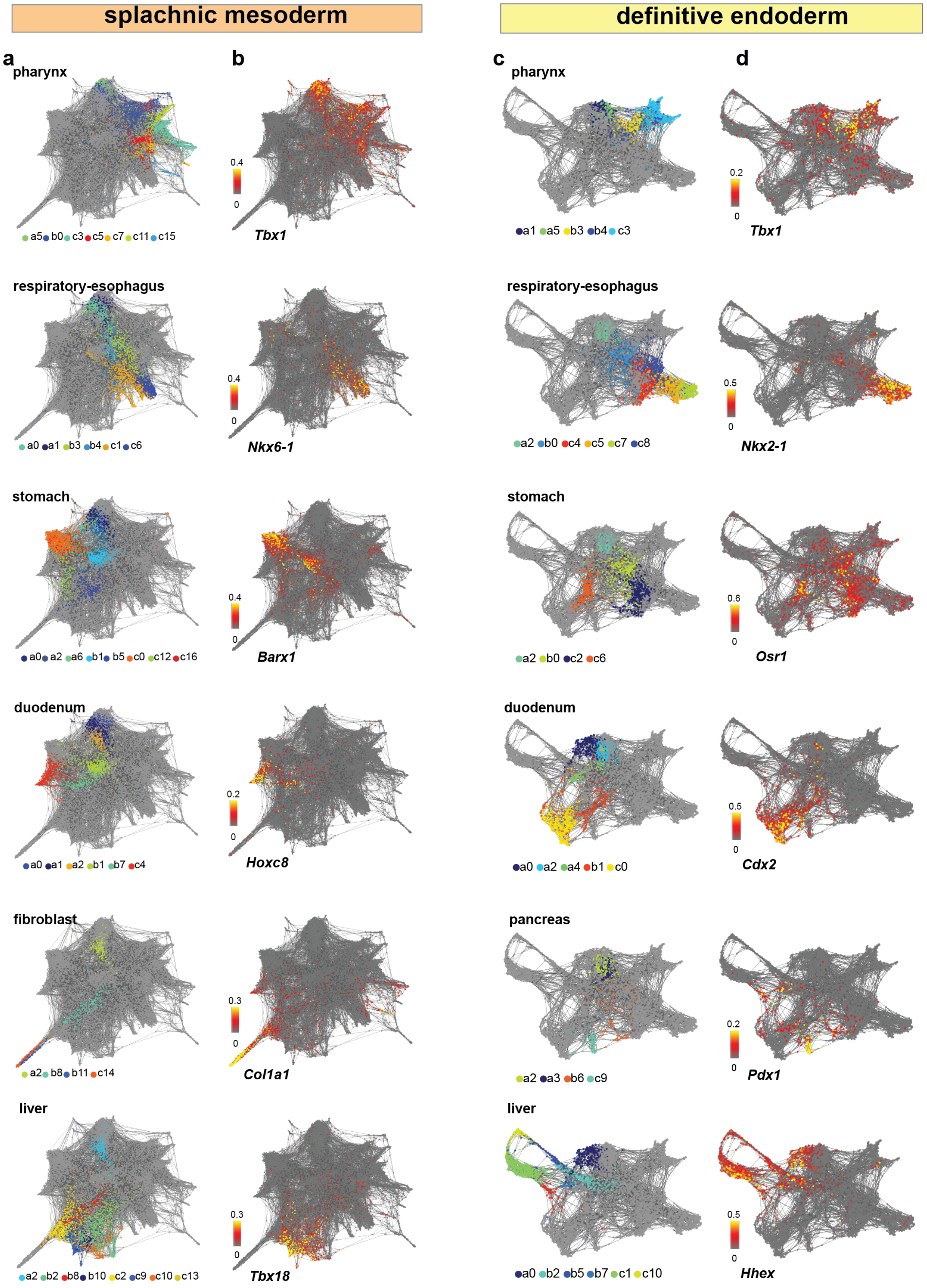

Supplementary Figure 6. SPRING plot of DE and SM cell trajectories. **a** and **b,** Spring plots of all SM cells (n=10,097) colored by **a**) stage specific lineage annotations and **b**) expression of key marker genes. **c** and **d,** Spring plots of all DE cells (n=4,448) colored by **c**) stage specific lineage annotations and **d**) expression of key marker genes. Gene expression is colored by normalized scaled expression in each cell. E8.5 cells = a_clusters, E9.0 cells = b_ clusters and E9.5 = c_clusters. Relevant to Fig. 3.

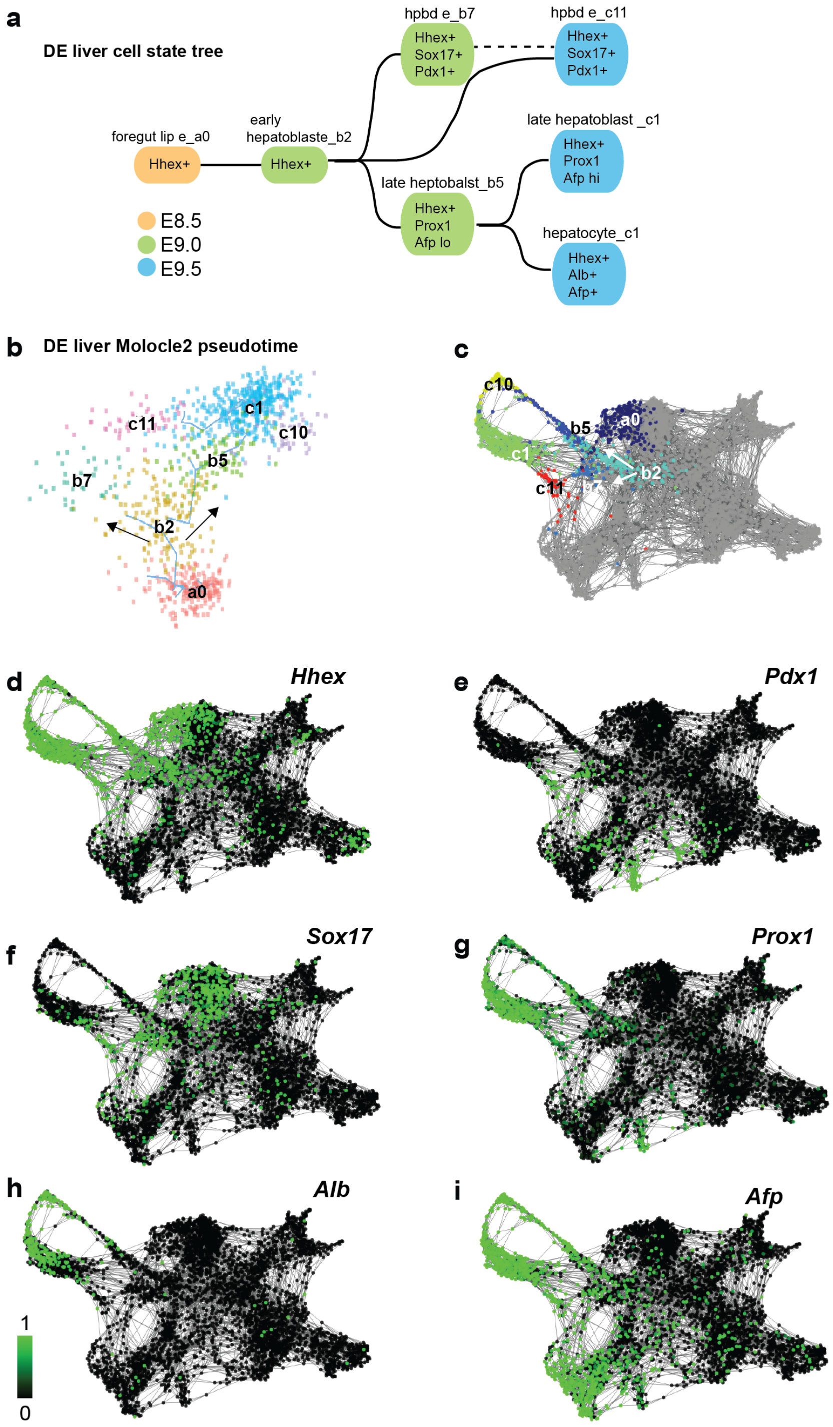

Supplementary Figure 7. Hepatic endoderm development. a, Cell state tree of the hepatic endodermal lineage with key marker genes indicated in each cell state. **b,** Pseudotime analysis of the hepatic DE lineage using Molocle_v3 suggests that at E9.0, the e_b2 cluster (early hepatoblasts) is a common progenitor of e_b5 (later hepatoblasts) and e_b7 (hepatopancreatic duct progenitors). E8.5 cells = a_clusters, E9.0 cells = b_ clusters and E9.5 = c_clusters **c-i**. SPRING plot with hepatic endodermal clusters colored by **c**) stage specific lineage annotations and **d-i**) expression of key marker genes. Gene expression is colored by normalized scaled expression in each cell. Relevant to Fig. 3.

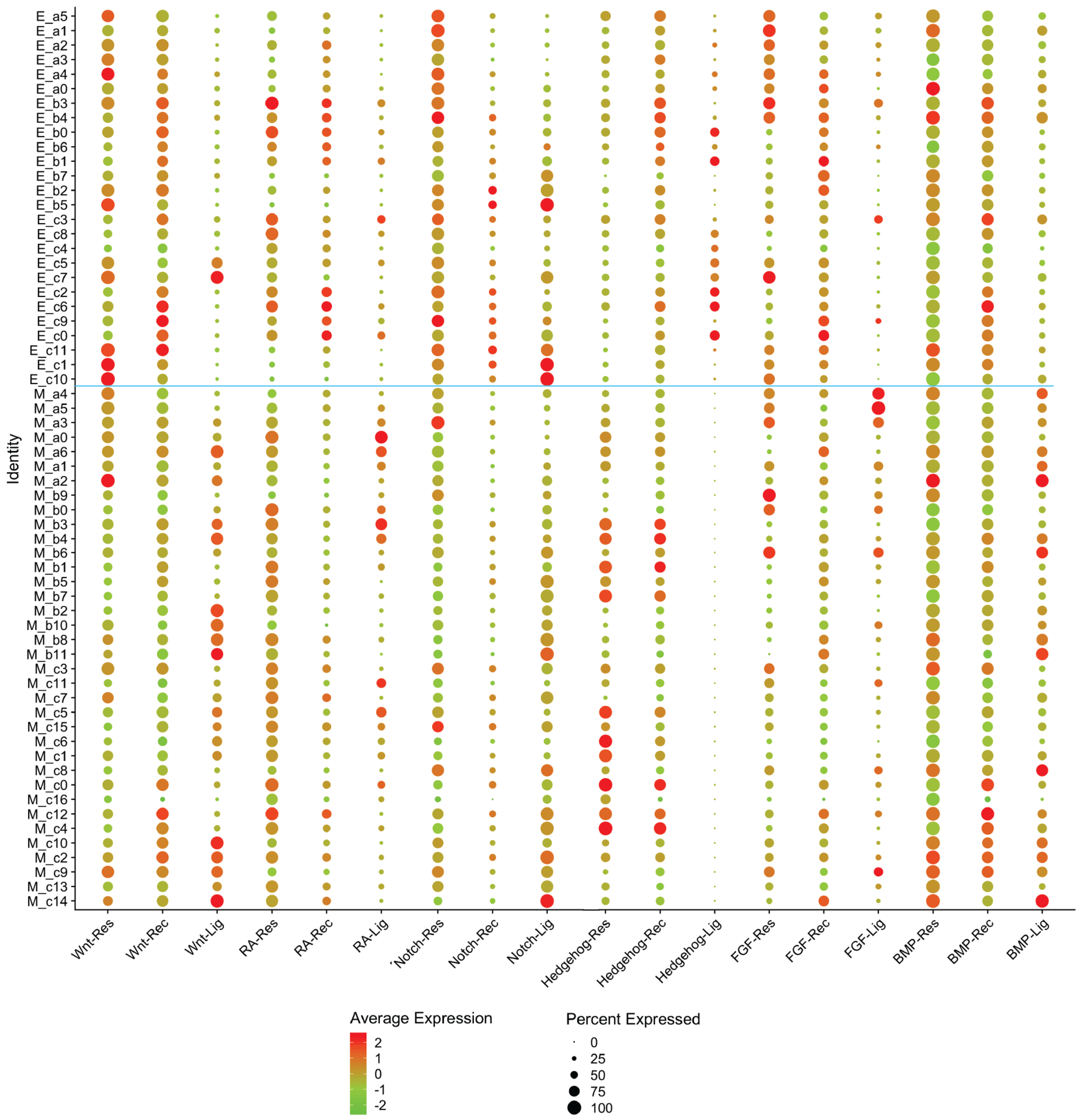

Supplementary Figure 8. Metagene expression for all ligands, receptors and context-independent response genes. Dot plot showing the average normalized expression of metagenes (X-axis) in each DE and SM cluster (Y-axis). For each cell signaling pathway (BMP, FGF, HH, Notch RA and canonical Wnt), we calculated a ligand-metagene, receptor-metagene and response-metagene by averaging the normalized expression of each individual gene for each pathway (e.g.: Wnt-ligand metagene =ΣWnt1+Wnt2+Wnt2b+Wnt3…Wnt10b expression/n) in each cell and cluster (see Methods for details). Color and size of the dot represents the normalized average metagene expression level in each cluster scaled from 2 to -2 and the % of expressing cell in each cluster. See Supplementary Data 2 for list of genes that make up each metagene and the numeric metagene expression data used to generate the plot. Relevant to Fig. 5.

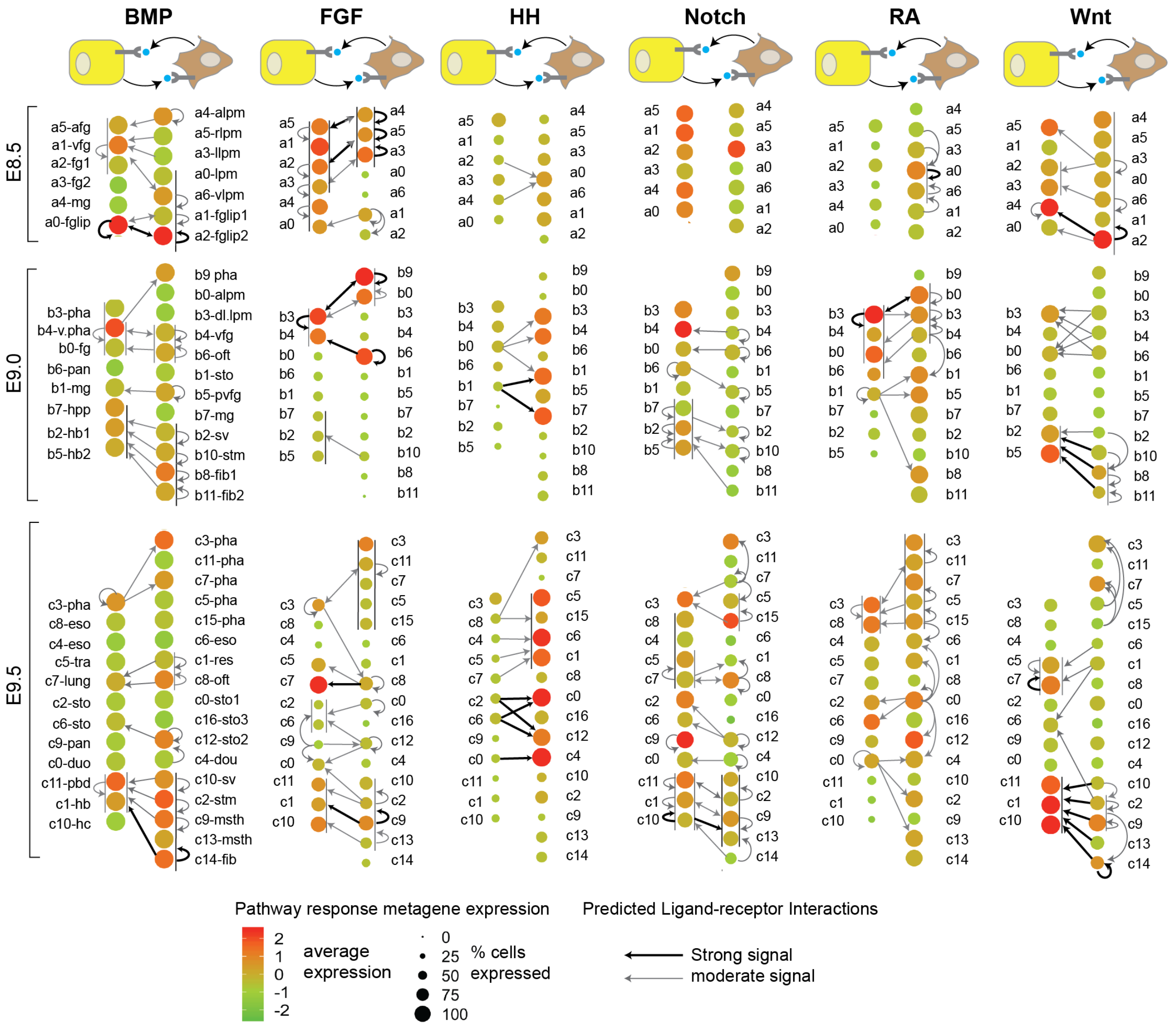

Supplementary Figure 9. Computationally predicted receptor-ligand interactions between different foregut cell populations. The schematics show paracrine signaling between the DE (yellow cells) and the SM (brown cells) for six major pathways. Below the schematics, DE and SM cell clusters of each stage are ordered along the A-P axis consistent with their location *in vivo*. Spatially adjacent DE and SM cell types are across from one another. Colored circles for each cluster indicate the likelihood that the cell population is responding to the signal based on the pathway response metagene expression levels (normalized and scaled from 2 to -2). Arrows represent the predicted source of the ligands showing paracrine and autocrine receptor-ligand pairs inferred from metagene expression profiles. Receptor-ligand pairing (arrows) were restricted to cell populations in close spatial proximity (see Methods for details). Thin vertical lines next to a group of clusters indicate different cell populations in spatial proximity that are all responding similarly. See Supplementary Data 2 for list of genes that make up each metagene and the numeric metagene expression data used to generate the plot. Relevant to Fig. 5.

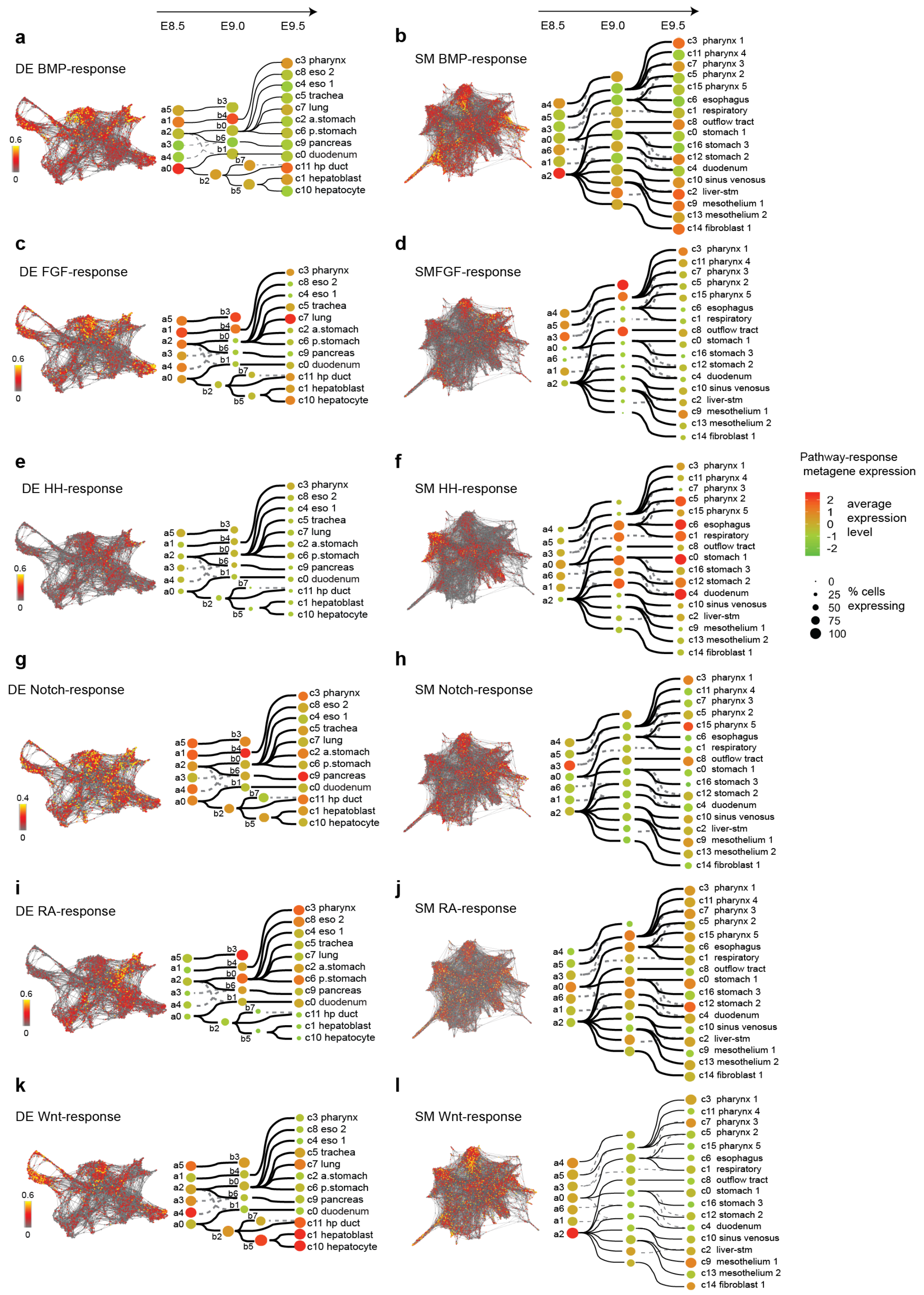

Supplementary Figure 10. Predicted temporal and spatial dynamics of signaling responses. a-i, Expression levels of the pathway-response metagene projected onto the DE and SM SPRING plots and cell state trees for the BMP (a-b), FGF (c-d), HH (e-f), Notch (g-h), RA (i-j) and canonical Wnt (k-l) pathways. This shows how coordinated spatial domains of signaling activity corresponding to cell lineages, are predicted to change over 24 hours from E8.5 – E9.5. Colored circles for each cluster indicate the likelihood that the cell population is responding to the signal based on the pathway response metagene expression levels (normalized and scaled from 2 to -2). Spring plots show the normalized scaled expression in each cell. Relevant to Fig. 5.

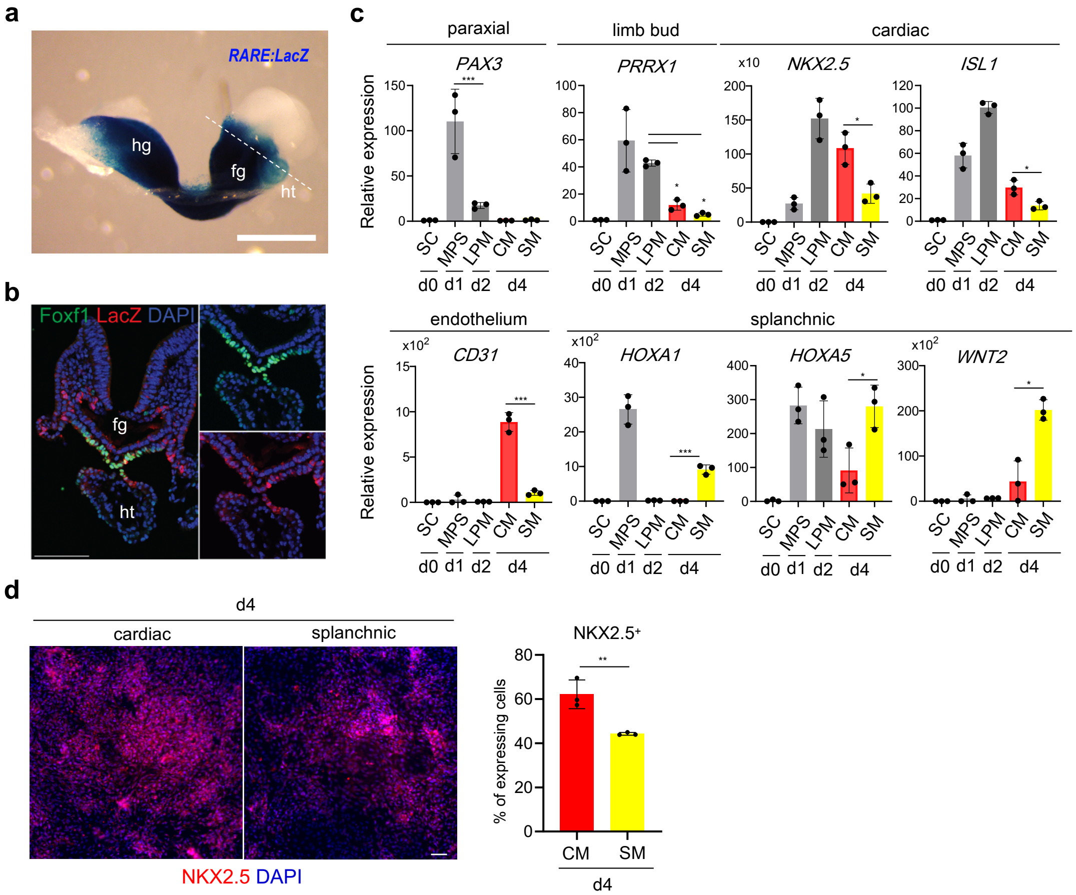

Supplementary Figure 11. RA suppresses cardiac mesoderm and promotes splanchnic mesoderm progenitors. **a.** Staining of *RARE-lacZ* transgenic mouse embryos confirms the single cell RNA-seq predictions that RA activity is higher in the splanchnic, than the cardiac mesenchyme at E8.5. Scale bar: 500µm. n = 3/3 embryos. **b,** Immunostaining of transvers section of *RARE-lacZ* transgenic mouse embryos. Right panels show higher magnification of separate channels. Scale bar: 100µm. n = 2/2 embryos. **c.** d4 PSC-derived SM cultures assayed by RT-PCR for paraxial mesoderm (*PAX3*), limb bud (*PRRX1*), cardiac mesoderm (*NKX2.5, ISL1*), endothelium (*CD31*), and SM (*HOXA1, HOXA5, WNT2*) markers. Histograms show the means ± S.D. n=3 independent biological samples. Similar results were obtained in at least two independent experiments. Statistical significance was calculated by a two-sided student's t-test (for *PAX3, NKX2.5, ISL1, CD31, HOXA1, HOXA5, WNT2*) or Dunnett's multiple comparisons test (for *PRRX1*). *p<0.05, **p<0.005, ***p<0.0005. Exact p-values were provided in Source data file. **d.** Immunostaining of d4 CM and SM cultures. Scale bar; 50µm. Histogram on the right show a quantification of NKX2-5+ cells, means ± S.D. Three independent fields were quantified. Statistical significance was calculated by a two-sided student's t-test. **p<0.009. Similar results were obtained in two independent experiments. fg; foregut, hg; hindgut, ht; heart, SC; Stem Cell, MPS; Middle Primitive Streak, CM, Cardiac Mesoderm, SM; Splanchnic mesoderm. Source data are provided in the Source Data file. Relevant to Fig. 7.

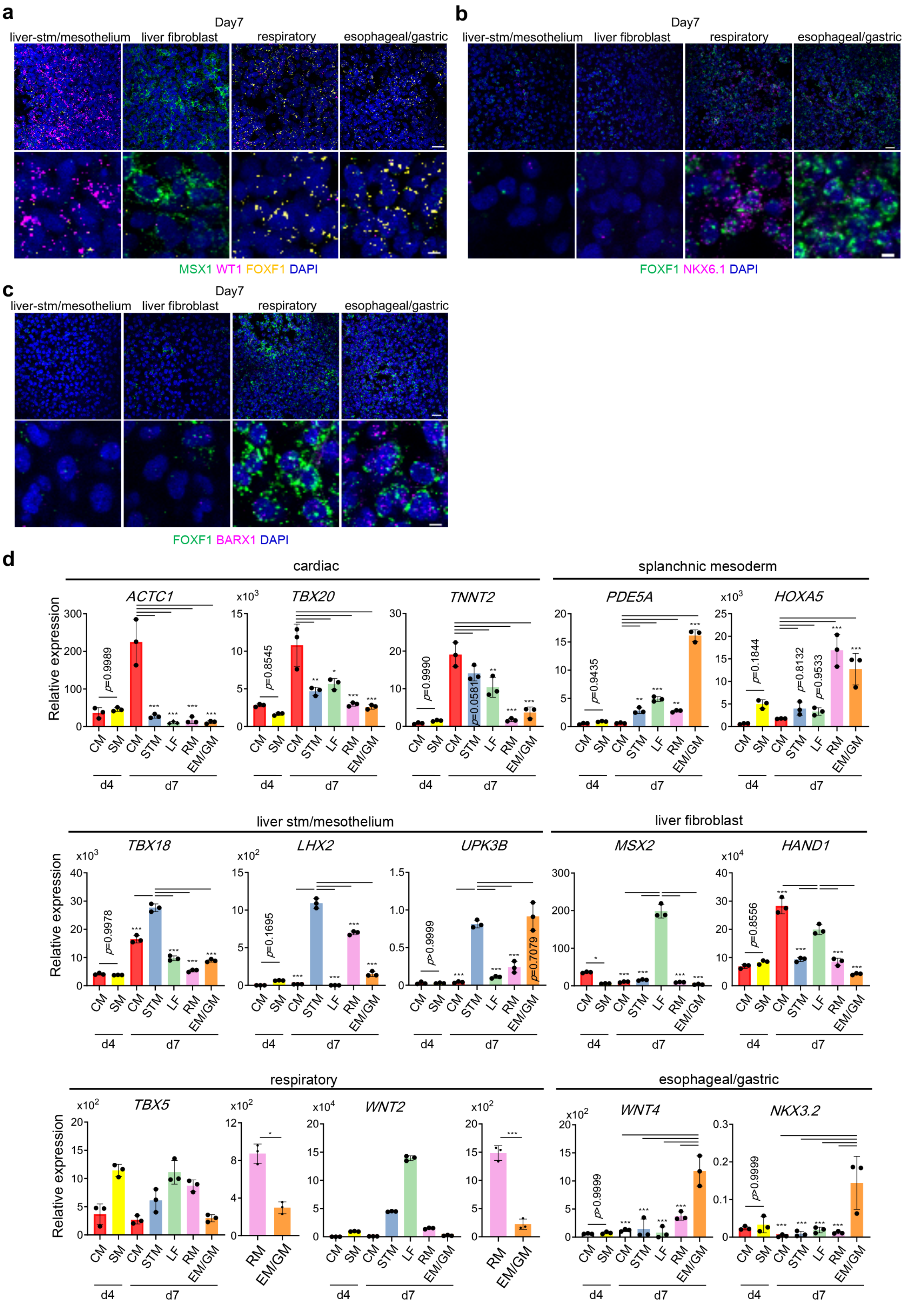

a
Day7
liver-stm/mesothelium
liver fibroblast
respiratory
esophageal/gastric
MSX1 WT1 FOXF1 DAPI
b
Day7
liver-stm/mesothelium
liver fibroblast
respiratory
esophageal/gastric
FOXF1 NKX6.1 DAPI
c
Day7
liver-stm/mesothelium
liver fibroblast
respiratory
esophageal/gastric
FOXF1 BARX1 DAPI
d
cardiac
splanchnic mesoderm
ACTC1
TBX20
TNNT2
PDE5A
HOXA5
Relative expression
p=0.9989
p=0.8545
p=0.9990
p=0.0581
p=0.9435
p=0.1844
p=0.8132
p=0.9533
CM
SM
STM
LF
RM
EM/GM
d4
d7
liver stm/mesothelium
liver fibroblast
TBX18
LHX2
UPK3B
MSX2
HAND1
p=0.9978
p=0.1695
p>0.9999
p=0.7079
p=0.8556
respiratory
esophageal/gastric
TBX5
WNT2
WNT4
NKX3.2
p>0.9999

Supplementary Figure 12 Additional analysis of d7 SM-like PSC cultures.
a-c, RNA-scope *in situ* analysis of different d7 SM-like cultures. Representative views of a single RNA-scope experiment validated the RT-PCR and Immunostaining analysis.
Scale bar 50μm (Upper panels), 10μm (Lower panels) (quantification is shown in Fig. 7d) **d,** RT-PCR analysis of mesoderm subtype markers based on the mouse scRNA-seq data; cardiac (*ACTC1*, *TBX20, TNNT2*), early SM (*PDE5A, HOXA5*); liver-stm/mesothelium (*TBX18, LHX2, UPK3B*), liver-fibroblast (*MSX2, HAND1*), esophageal/gastric (*WNT4, NKX3-2*). Histograms show the means ± S.D (n=3 biologically independent samples). Experiments were repeated at least twice with similar results. Statistical significance was calculated using a two-sided Tukey's multiple comparisons test. *p<0.05, **p<0.005, ***p<0.0005. Exact p-values were provided in Source data file. SC; Stem Cell, MPS; Middle Primitive Streak, CM, Cardiac Mesoderm, SM; Splanchnic Mesoderm, STM; Septum Transversum Mesenchyme, LF; Liver Fibroblast, RM Respiratory Mesenchyme, EM/GM; Esophageal/Gastric Mesenchyme. Source data for the graphs are provided in the Source Data file. Relevant to Fig. 7.

Supplementary Table 1. Information on antibodies (top table) and RT-PCR primers used in this study (lower table).

antibody	company	species	dilution	catalog #
β-gal	Abcam	chicken	1000	ab9361
CDH1	R&D system	rat	1000	MAB7481
FOXA2	Santa Cruz	goat	500	sc-6554
FOXF1	R&D	goat	500	AF4798
NKX6-1	DSHB	mouse	100	F55A10
WT1	Abcam	rabbit	300	ab89901
Nkx2-5	Santa Cruz	goat	200	sc-8697
NKX6-1	DSHB	mouse	100	F55A12
Troponin	Thermo NeoMarkers	mouse	200	clone 13-11
p-Smad1,5,8	Millipore	rabbit	500	AB3848
Sox2	Abcam	mouse	200	ab79351
Nkx2-1	Santa Cruz	rabbit	200	sc-13040x

Gene	Forward Primer	Reverse Primer
ACTC1	CTTCATTGGTATGGAATCTGCTG	TACTCTTGCTTGCTAATCCAC
BARX1	CCAGTGGGAACTTGAACACC	CTGAAGTTCGGCGTGCAG
FOXF1	AGCAGCCGTATCTGCACCAGAA	CTCCTTTCGGTCACACATGCTG
HAND1	GTGCGTCCTTTAATCCTCTTC	GTGAGAGCAAGCGGAAAAG
HOXA1	GTACG CTACCTGGGTCAAC	ACTTGGGTCTCGTTGAGCTG
HOXA5	CGCCCAACCCCAGATCTAC	CGGGCCGCCTATGTTGT
ISL1	AGATTATATCAGGTTGTACGGGATCA	ACACAGCGGAAACACTCGAT
LHX2	TCGGGACTTGGTTTATCACCT	GCAAGCGGCAGTAGACCAG
MSC	TATGAGAACGGCTACGTGCAC	AGTCCGATTTAAGCGGTGGTT
MSX1	CTCCGCAAACACAAGACGAAC	GGCGGTTCTGGAACCATATCT
MSX2	CGCCAAGACATATGAGCCCT	GTTCTGCCTCCTGCAGTCTT
NKX2.5	TGGAGAAGACAGAGGCGGACAA	ATAGACCTGCGCCTGCGAGAA
NKX3.2	CAACACCGTCGTCCTCG	CCGCTTCCAAAGACCTAGAG
NKX6.1	ATGACAGAGAGTCAGGTCAAGG	CTCCGAGTCCTGCTTCTTCTT
PAX3	GCCGCATCCTGAGAAGTAAA	CTTCATCTGATTGGGGTGCT
PDE5A	GCAGAGTCCTCGTGCAGATAA	GTCTAAGAGGCCGGTCAAATTC
PRRX1	TGCAGGCTTTGGAGCGTGTCTT	CTCATTCCTGCGGAACTTGGCT
TBX5	ACAAAGTGAAGGTGACGGGCCTTA	ATCTGTGATCGTCGGCAGGTACAA
TBX18	GACGATCTTTCTCCCATCAAGC	CTATCTTCAGGCGAGTAATCTGC
TBX20	GGCGACGGAGAACACAATCAA	CTGGGCACAGGACGACTTC
TNNT2	TTCACCAAAGATCTGCTCCTCGCT	TTATTACTGGTGTGGAGTGGGTGTGG
UPK1B	TGGAAGCAACGAACAGTTGA	CTACCGTGTGCGCAGAAA
UPK3B	ATACCGTCTGGCTCGTGGTG	CAGGGGCAGCGTCATGTAGT
WNT2	CTGTATCAGGGACCGAGAGG	CCCACAGCACATGACTTCAC
WNT4	CCTTCTCACAGTCGTTTG	CACAGCCGTCGATGGCCTT
WT1	ATAGGCCAGGGCATGTGTATGTGT	AGTTGCCTGGCAGAACTACATCCT

Supplementary Table 2. RNA-scope probes for *in situ* hybridization of mouse frozen section (top table) and human adherent PSC cultures (lower table).

Mouse Gene	Probe Name,<br>Catalog Number	Fluorepore
Barx1	RNAscope® Probe- Mm-Barx1<br>414681	Opal690 (1:1000)<br>40˚C, 30min
Foxf1	RNAscope® Probe- Mm-Foxf1<br>473058	Opal520 (1:1000)<br>40˚C, 45min
Gata4	RNAscope® Probe- Mm-Gata4<br>417881	Opal520 (1:1000)<br>40˚C, 45min
Hand1	RNAscope® Probe- Mm-Hand1-C2<br>429651-C2	Opal570 (1:1000)<br>40˚C, 30min
Hand2	RNAscope® Probe- Mm-Hand2<br>499821	Opal520 (1:1000)<br>40˚C, 45min
Msx1	RNAscope® Probe- Mm-Msx1<br>421841	Opal520 (1:1000)<br>40˚C, 45min
Nkx6.1	RNAscope® Probe- Mm-Nkx6-1-C3<br>473061-C3	Opal690 (1:1000)<br>40˚C, 30min
Wnt2	RNAscope® Probe- Mm-Wnt2-C2<br>313601-C2	Opal570 (1:1000)<br>40˚C, 30min
Wnt4	RNAscope® Probe- Mm-Wnt4-C2<br>401101-C2	Opal570 (1:1000)<br>40˚C, 30min
Wt1	RNAscope® Probe- Mm-Wt1-C2<br>432711-C2	Opal570 (1:1000)<br>40˚C, 30min

Human Gene	Probe Name,<br>Catalog Number	Fluorepore
BARX1	RNAscope® Probe- Hs-BARX1<br>432981	Opal520 (1:1000)<br>40˚C, 45min
FOXF1	RNAscope® Probe- Hs-FOXF1-C3<br>505741-C3	Opal690 (1:1000)<br>40˚C, 30min
MSX1	RNAscope® Probe- Hs-MSX1<br>470701	Opal520 (1:1000)<br>40˚C, 45min
NKX6.1	RNAscope® Probe- Hs-NKX6-1-C2<br>450541-C2	Opal570 (1:1000)<br>40˚C, 30min
WT1	RNAscope® Probe- Hs-WT1-C2<br>415581-C2	Opal570 (1:1000)<br>40˚C, 30min
