# Supplementary material for: Single cell transcriptomics identifies a signaling network coordinating endoderm and mesoderm diversification during foregut organogenesis
Source: Nat Commun. 2020 Aug 27;11:4158. doi: 10.1038/s41467-020-17968-x (PMC7453027; doi:10.1038/s41467-020-17968-x)
Supplement: Supplementary file 4 — Reporting Summary [file 41467_2020_17968_MOESM4_ESM.pdf]

nature research

Corresponding author(s): Aaron Zorn

Last updated by author(s): Jun 18, 2020

Reporting Summary

Nature Research wishes to improve the reproducibility of the work that we publish. This form provides structure for consistency and transparency in reporting. For further information on Nature Research policies, see our Editorial Policies and the Editorial Policy Checklist.

Statistics

For all statistical analyses, confirm that the following items are present in the figure legend, table legend, main text, or Methods section.

n/a	Confirmed	
☐	☒	The exact sample size (*n*) for each experimental group/condition, given as a discrete number and unit of measurement
☐	☒	A statement on whether measurements were taken from distinct samples or whether the same sample was measured repeatedly
☐	☒	The statistical test(s) used AND whether they are one- or two-sided<br>*Only common tests should be described solely by name; describe more complex techniques in the Methods section.*
☒	☐	A description of all covariates tested
☒	☐	A description of any assumptions or corrections, such as tests of normality and adjustment for multiple comparisons
☐	☒	A full description of the statistical parameters including central tendency (e.g. means) or other basic estimates (e.g. regression coefficient) AND variation (e.g. standard deviation) or associated estimates of uncertainty (e.g. confidence intervals)
☐	☒	For null hypothesis testing, the test statistic (e.g. *F*, *t*, *r*) with confidence intervals, effect sizes, degrees of freedom and *P* value noted<br>*Give P values as exact values whenever suitable.*
☒	☐	For Bayesian analysis, information on the choice of priors and Markov chain Monte Carlo settings
☒	☐	For hierarchical and complex designs, identification of the appropriate level for tests and full reporting of outcomes
☒	☐	Estimates of effect sizes (e.g. Cohen's *d*, Pearson's *r*), indicating how they were calculated

Our web collection on statistics for biologists contains articles on many of the points above.

Software and code

Policy information about availability of computer code

Data collection	Single cell transcriptomes were generated on Chromium Single Cell Controller instrument (10x Genomics) and sequenced in an Illumina HiSeq 2500.Embryo images were collected on Nikon confocal microscope (https://www.microscope.healthcare.nikon.com/products/confocal-microscopes) or a Leica MZ10F with software Leica Application Suite [v4.9]. Figure were generated using Adobe Illustrator and Photoshop suite [v2015].
Data analysis	We used the following software for data analysis: R [v>=3.4.4], Perl [v5.18], python [v2.7], Seurat [v2.3.4, v3.0.0], Monocle [v3.0], SPRING [v1.0], URD [v1.0], cellranger [v2.0.0], CSBB [v3.0], GSEA [v3.0], Prisim [v8] and Imaris [v9.2]. Custom codes/algorithms are available on GitHub [https://github.com/ZornLab/Single-cell-transcriptomics-reveals-a-signaling-roadmap-coordinating-endoderm-and-mesoderm-lineage]

For manuscripts utilizing custom algorithms or software that are central to the research but not yet described in published literature, software must be made available to editors and reviewers. We strongly encourage code deposition in a community repository (e.g. GitHub). See the Nature Research guidelines for submitting code & software for further information.

Data

Policy information about availability of data

All manuscripts must include a data availability statement. This statement should provide the following information, where applicable:

- Accession codes, unique identifiers, or web links for publicly available datasets
- A list of figures that have associated raw data
- A description of any restrictions on data availability

We have included a data availability statement. The scRNA-seq and bulk RNA-seq data (including bam, raw counts and cell annotations) are available at Gene Expression Omnibus (GEO): GSE136689 (https://www.ncbi.nlm.nih.gov/geo/query/acc.cgi?acc=GSE136689) and GSE136687 (https://www.ncbi.nlm.nih.gov/geo/query/acc.cgi?acc=GSE136687). All other data is freely available either in the manuscript, Supplementary Information or Source Datafiles. All the code [scripts, R-

packages and software's] and their documentation is available on https://github.com/ZornLab/Single-cell-transcriptomics-reveals-a-signaling-roadmap-coordinating-endoderm-and-mesoderm-lineage.We have established an open access R-Shinny webapp, where readers can explore the data: https://research.cchmc.org/ZornLab-singlecell

Field-specific reporting

Please select the one below that is the best fit for your research. If you are not sure, read the appropriate sections before making your selection.

☒ Life sciences ☐ Behavioural & social sciences ☐ Ecological, evolutionary & environmental sciences

For a reference copy of the document with all sections, see nature.com/documents/nr-reporting-summary-flat.pdf

Life sciences study design

All studies must disclose on these points even when the disclosure is negative.

Sample size	No statistical methods were used to predetermine sample size. The sample sizes were chosen based on previous reports of single cell RNA sequencing analysis with mouse embryos. We used the maximum number of embryos that could be dissected in the 2 hour window of tissue viability. We pooled dissected foregut tissue from 16 embryos at E8.5 (3 litters) , 20 embryos at E9.0 (3 litters), 18 embryos for anterior E9.5 and 15 embryos for posterior E9.5 (two litters). This resulted in cell suspensions sufficient to obtain >9,000 single cell transcriptomes for each time point. This is comparable if not larger than most single cell studies to date and sufficient to identify various cell clusters described in the manuscript. For bulk RNA-seq foregut tissue was dissected from E9.5 double mutant Gli2-/-;Gli3-/- (n=3) and Gli2+/-;Gli3+/- heterozygous litter mate controls (n=3). This was based on the number of biological replicates necessary for robust statistical analysis of differential expressed transcripts with the limitation of 1/16 embryos being double homozygous. For other embryo experiments at least two embryos (more typically 3-5) were used for each assay.
Data exclusions	We used pre-established criteria in the single cell analysis field to exclude low quality cells as detailed below: barcodes with less than ~5k UMI counts were not included in downstream analysis. Basic filtering was carried out where all genes expressed >= 3 cells and all cells with at least 100 detected genes were included. QC was based on nGene and percent.mito parameters to remove the multiplets and cells with high mitochondrial gene expression using the following parameters for each sample [E8.5: [ngene(-Inf,7500), percent.mito(-Inf,0.15)], E9.0: [ngene (-Inf,7000), percent.mito(-Inf,0.15)] and E9.5: [ngene(-Inf,7500), percent.mito(-Inf,0.15)]]. For other experiments no data or samples were excluded.
Replication	For single cell sequencing, we pooled dissected foregut tissue from 16 embryos at E8.5 , 20 embryos at E9.0, 18 embryos for anterior E9.5 and 15 embryos for posterior E9.5, isolated from 2-3 litters for each time point. For bulk RNA sequencing, three biological replicates are included for each genotype (each control and mutant embryos were from separate litters). For gene expression validations using in situ or immunostaining, two or more biological samples were used for each gene expression analysis For human pluripotent stem cell differentiation, experiments were repeated more than five times in WA01- H1 hES cells and twice in iPSC72_3 cell lines. All attempts at replication were successful.
Randomization	For the single cell RNA-seq analysis all cells from all three stages were processed both non-randomized (each stage separately) and randomized (cells from all stages combined) with very similar results. For other mouse experiments embryos were randomized during analysis and with litter mates used as controls. No other randomizations were performed as this was not a case-control study and we were documenting gene expression in normal embryos.
Blinding	Embryo collection, RNA library preparation and sequencing were performed by different researchers and they were blinded during the process. Cell identify and stage was blinded during the single cell RNA-seq analysis. Investigator were not blinded to experimental groups or genotype for other experiments after processing as this would preclude grouping of biological replicates for analysis.

Reporting for specific materials, systems and methods

We require information from authors about some types of materials, experimental systems and methods used in many studies. Here, indicate whether each material, system or method listed is relevant to your study. If you are not sure if a list item applies to your research, read the appropriate section before selecting a response.

Materials & experimental systems

n/a	Involved in the study
☐	☒ Antibodies
☐	☒ Eukaryotic cell lines
☒	☐ Palaeontology and archaeology
☐	☒ Animals and other organisms
☒	☐ Human research participants
☒	☐ Clinical data
☒	☐ Dual use research of concern

Methods

n/a	Involved in the study
☒	☐ ChIP-seq
☒	☐ Flow cytometry
☒	☐ MRI-based neuroimaging

Antibodies

Antibodies used	antibody company species dilution catalog #<br>β-gal Abcam chicken 1000 ab9361<br>CDH1 R&D system rat 1000 MAB7481<br>FOXA2 Santa Cruz goat 500 sc-6554<br>FOXF1 R&D goat 500 AF4798<br>Nkx2-1 Santa Cruz rabbit 200 sc-13040x<br>Nkx2-5 Santa Cruz goat 200 sc-8697<br>NKX6-1 DSHB mouse 100 F55A12<br>p-Smad1,5,8 Millipore rabbit 500 AB3848<br>Sox2 Abcam mouse 200 ab79351<br>WT1 Abcam rabbit 300 ab89901
Validation	β-gal: Dev Cell. 2019 49:293-300.<br>CDH1: J Bone Miner Res, 2016;0(0):; Sci Rep, 2016;6(0):37914.<br>FOXA2: Cell Rep. 2016 Jun 28;16(1):66-78.<br>FOXF1: Cell Rep, 2018;23(2):442-458.<br>Nkx2-1: J Neurosci. 2015 Apr 29;35(17):6667-88.<br>Nkx2-5: J. Biol. Chem. 278: 23807-23816 (2013)<br>NKX6-1: J Histochem Cytochem. 2006 May;54(5):567-74.<br>p-Smad1,5,8: Cell Rep. 2016 Jun 28;16(1):66-78.<br>Sox2: Sci Rep 10:148 (2020)..<br>WT1: Dev Biol 445:178-188 (2019).<br>We performed additional validation test on mouse tissue that was known based on RNA-expression to either be positive or negative for each target protein.

Eukaryotic cell lines

Policy information about cell lines

Cell line source(s)	Two hPSC lines were used in this study; 1) WAOl-Hl human embryonic stem cells purchased from WiCell (NIH approval number NIHhESC-10-0043 and NIHhESC-10-0062) and 2) human iPSC72_3 generated by the CCHMC Pluripotent Stem Cell Facility.
Authentication	Both cell lines have been authenticated as follows: i) Cell identity; via STR profiling by Genetica DNA Laboratory (a LabCorp brand; Burlington, NC), ii) Genetic stability; by standard metaphase spread and G-banded karyotype analysis in CCHMC Cytogenetics Laboratory and iii) Functional pluripotency; cells were subjected to analysis of functional pluripotency by teratoma assay demonstrating ability to differentiate into each of the three germ layer.
Mycoplasma contamination	Both cell lines routinely tested negative for mycoplasma contamination.
Commonly misidentified lines<br>(See ICLAC register)	No commonly misidentied lines were used in this study.

Animals and other organisms

Policy information about studies involving animals; ARRIVE guidelines recommended for reporting animal research

Laboratory animals	Mice of either C57BL6 (for scRNA-seq) or mixed background strains (all other experiments), ranging from 1 month to 12 months of age, were used in this study. Mice were housed at 72oF, 30-70% humidity and a 14/10 hour light/dark cycle. Males and females were both included of roughly equal ratio. Gli2+/-;Gli3+/- mice were maintained on a mixed genetic background. Timed matings were used to obtain embryos harvested at embryonic day E8.5-9.5 as indicated. Embryo sex was not determined.
Wild animals	The study did not use any wild animals
Field-collected samples	The study did not involve samples collected from the field
Ethics oversight	All mouse experiments were performed in accordance with protocols approved by the Cincinnati Children's Hospital Medical Center Institutional Animal Care and Use Committee (IACUC).

Note that full information on the approval of the study protocol must also be provided in the manuscript.
