# Supplementary material for: Single cell transcriptomics identifies a signaling network coordinating endoderm and mesoderm diversification during foregut organogenesis
Source: Nat Commun. 2020 Aug 27;11:4158. doi: 10.1038/s41467-020-17968-x (PMC7453027; doi:10.1038/s41467-020-17968-x)
Supplement: Supplementary file 5 — Description of Additional Supplementary Files [file 41467_2020_17968_MOESM5_ESM.pdf]

Title: Supplementary Data 1.
Description: Highly variable marker genes for each DE and SM cluster. **a.** Top distinguishing marker genes for endoderm clusters (excel file tab-1). **b,** Top distinguishing marker genes for splanchnic mesoderm clusters (tab-2). **c**, Transcription factors with enriched expression in endoderm clusters (tab-3). **d**, Transcription factors with enriched expression in splanchnic mesoderm clusters (tab-4). Differential Markers and TFs were obtained using a one-sided Wilcoxon rank sum test in Seurat FindMarkers Function. Relevant to Figs. 1-4.

Title: Supplementary Data 2.
Description: Analysis of signaling pathway metagene profiles. **a.** List of BMP, FGF, HH, Notch and canonical Wnt pathway genes used to calculate metagene profiles (tab-1). **b.** Normalized and scaled average expression of each metagene in each DE and SM cluster (tab-2). **c**. Log2 average expression of metagene profiles (tab-3). **d.** Average Log2 counts of pathway genes in each cluster; BMP (tab-4), FGF (tab-5), HH (tab-6), Notch (tab-7), RA (tab-8) and Wnt (tab-9). Relevant to Fig. 5.

Title: Supplementary Data 3.
Description: Differentially expressed transcripts in the bulk RNA-sequencing of E9.5 mouse foregut comparing *Gli2-/-;Gli3-/-* to *Gli2+/-;Gli3+/-* littermates (GSE136687). n= 3 embryos / genotype. Differential transcripts were obtained using generalized linear model statistics in empirical analysis of digital gene expression (edgeR). Relevant to Fig. 6.

Title: Supplementary Movie 1.
Description: Whole-mount staining of mouse embryo foregut at E9.5. Foregut was stained for Nkx2.1 (*red*), Nkx6.1 (*green*), Foxa2 (*blue*), and DAPI (*grey*).

Title: Supplementary Movie 2.
Description: Whole-mount staining of mouse embryo foregut at E9.5. Foregut was stained for Foxf1 (*red*), Nkx6.1 (*green*), Cdh1 (*blue*), and DAPI (*grey*).
